# Performance of LBSap Vaccine after Intradermal Challenge with *L. infantum* and Saliva of *Lu. longipalpis*: Immunogenicity and Parasitological Evaluation

**DOI:** 10.1371/journal.pone.0049780

**Published:** 2012-11-26

**Authors:** Bruno Mendes Roatt, Rodrigo Dian de Oliveira Aguiar-Soares, Juliana Vitoriano-Souza, Wendel Coura-Vital, Samuel Leôncio Braga, Rodrigo Corrêa-Oliveira, Olindo Assis Martins-Filho, Andréa Teixeira-Carvalho, Marta de Lana, Nelder Figueiredo Gontijo, Marcos José Marques, Rodolfo Cordeiro Giunchetti, Alexandre Barbosa Reis

**Affiliations:** 1 Laboratório de Imunopatologia, Núcleo de Pesquisas em Ciências Biológicas, Universidade Federal de Ouro Preto, Ouro Preto, Minas Gerais, Brazil; 2 Laboratório de Imunologia Celular e Molecular, Instituto de Pesquisas René Rachou, Fundação Oswaldo Cruz, Belo Horizonte, Minas Gerais, Brazil; 3 Laboratório de Biomarcadores de Diagnóstico e Monitoração, Instituto René Rachou, Fundação Oswaldo Cruz, Belo Horizonte, Minas Gerais, Brazil; 4 Laboratório de Doença de Chagas, Núcleo de Pesquisas em Ciências Biológicas, Universidade Federal de Ouro Preto, Ouro Preto, Minas Gerais, Brazil; 5 Laboratório de Fisiologia de Insetos Hematófagos, Departamento de Parasitologia, Universidade Federal de Minas Gerais, Belo Horizonte, Minas Gerais, Brazil; 6 Laboratório de Biologia Molecular e Biotecnologia, Departamento de Biociências, Universidade Federal de Alfenas, Alfenas, Minas Gerais, Brazil; 7 Laboratório de Biologia das Interações Celulares, Departamento de Morfologia, Universidade Federal de Minas Gerais, Belo Horizonte, Minas Gerais, Brazil; 8 Departamento de Análises Clínicas, Escola de Farmácia, Universidade Federal de Ouro Preto, Ouro Preto, Minas Gerais, Brazil; London School of Hygiene and Tropical Medicine, United Kingdom

## Abstract

In the last decade, the search for new vaccines against canine visceral leishmaniasis has intensified. However, the pattern related to immune protection during long periods after experimental infection in vaccine trials is still not fully understood. Herein, we investigated the immunogenicity and parasitological levels after intradermal challenge with *Leishmania infantum* plus salivary gland extract in dogs immunized with a vaccine composed of *L. braziliensis* antigens plus saponin as an adjuvant (LBSap vaccine). The LBSap vaccine elicited higher levels of total anti-*Leishmania* IgG as well as both IgG1 and IgG2. Furthermore, dogs vaccinated had increased levels of lymphocytes, particularly circulating B cells (CD21^+^) and both CD4^+^ and CD8^+^ T lymphocytes. LBSap also elicited an intense *in vitro* cell proliferation associated with higher levels of CD4^+^ T lymphocytes specific for vaccine soluble antigen and soluble lysate of *L. infantum* antigen even 885 days after experimental challenge. Furthermore, LBSap vaccinated dogs presented high IFN-γ and low IL-10 and TGF-β1 expression in spleen with significant reduction of parasite load in this tissue. Overall, our results validate the potential of LBSap vaccine to protect against *L. infantum* experimental infection and strongly support further evaluation of efficiency of LBSap against CVL in natural infection conditions.

## Introduction

Visceral leishmaniasis (VL), caused by *Leishmania infantum* (syn. *Leishmania chagasi*), is the most severe and fatal form of leishmaniasis if left untreated once symptoms begin. Dogs are extremely susceptible to infection and are considered the most important domestic reservoirs of the parasites [1], [2]. Thus, canine visceral leishmaniasis (CVL) represents a serious public health problem in both human and veterinary medicine in various areas of the globe including regions of the Americas, the Mediterranean basin, Asia and Europe [3]. Often, the prevalence of infected dogs in endemic areas is high [4], with animals developing variable clinical signs of infection but also subclinical cases. In addition, subclinically infected dogs and symptomatic animals are important reservoirs for the infection of sandflies [5], [6]. The current control strategy to interrupt transmission includes systematic treatment of human cases, vector control and the elimination of seropositive dogs [3], [7], [8]. Recently an increase of drug resistance, toxicity of chemotherapy and difficulties in epidemiological control has been documented [9], [10], [11]. Therefore, the development of a vaccine against CVL for controlling canine disease and reducing the incidence of human VL is fundamental and urgent [7].

Significant efforts are being made by several groups to develop a vaccine against CVL. Lemesre *et al*. [12], using a second-generation vaccine containing *L. infantum* secreted/excreted proteins in a formulation with muramyldipeptide - MDP, obtained protection in beagles, with 75% efficacy (92% of protection) in an endemic area of France after 2 years of follow-up [12], [13]. In contrast, recombinant vaccines like the multicomponent Leish-111f fusion protein associated with different adjuvants (MPL-SE or AdjuPrime) showed significant immunogenic results in dogs after challenge with *L. infantum* [14] but failed to prevent natural *L. infantum* infection and the progression of the disease in an open kennel trial [15]. In addition, a vaccine composed of a glycoproteic complex, fucose–mannose ligand, associated with saponin (Leishmune®/Fort Dodge) showed 76% to 80% vaccine efficacy in dogs in a field assay in an endemic area in Brazil [16], [17], [18]. Another vaccine licensed in Brazil, composed of the A2 recombinant protein in formulation with saponin (Leish-Tec®/Hertape Calier), was also tested in a kennel assay (Phase I and II) and presented high levels of specific antibodies with positive parasitism in the bone marrow of four of seven vaccinated dogs after 9 months post experimental challenge with *L. infantum* [19].

Considering their wide spectrum of antigenicity, cost and safety, vaccines composed of crude antigens also represent an excellent tool for immunoprophylaxis and control of CVL in endemic areas [20], [21], [22], [23], [24], [25], [26], [27]. Our research group has developed a vaccine against CVL composed of *L. braziliensis* antigens plus saponin as an adjuvant (LBSap vaccine). In previous studies the LBSap vaccine was shown to be safe for use, with no ulcerated lesions being observed at the inoculation sites [23], [28]. We also demonstrated the vaccine’s immunogenicity, including an increase in immunoglobulin isotypes; higher levels of T lymphocytes, particularly circulating CD8^+^ T lymphocytes; intense cell proliferation; NO production during *in vitro* stimulation; and higher levels of *Leishmania*-specific CD8^+^ T lymphocytes after three doses in dogs [23]. Considering the promising results of the LBSap vaccine, the aim of this study was to further evaluate the immunogenicity and perform parasitological analysis after challenge by intradermal inoculum with *L. infantum* plus salivary gland extract (SGE) of *Lutzomyia longipalpis* 885 days later. We also evaluated the cellular immune response employing real-time PCR for determine the cytokines profile and parasite burdens in the spleen.

## Materials and Methods

The study protocol was approved by the Ethical Committee for the Use of Experimental Animals of the Universidade Federal de Ouro Preto, Ouro Preto - MG, Brazil.

### Study Animals, Vaccination and Experimental Challenge

In this study we used LBSap vaccine as previously described by Giunchetti *et al*. [23] and registered at the Instituto Nacional da Propriedade Industrial (patent: PI 0601225-6, Rio de Janeiro, RJ, Brazil) composed of *L. braziliensis* crude antigens and saponin as an adjuvant. Twenty male and female mongrel dogs were born and reared in the kennels of the Animal Science Center, Universidade Federal de Ouro Preto, Ouro Preto, Minas Gerais, Brazil. The dogs were treated at 7 months with an anthelmintic and vaccinated against rabies (Tecpar, Curitiba-PR, Brazil), canine distemper, type 2 adenovirus, coronavirus, parainfluenza, parvovirus and leptospira (Vanguard® HTLP 5/CV-L; Pfizer Animal Health, New York, NY, USA). The absence of specific anti-*Leishmania* antibodies was confirmed by indirect fluorescence immunoassay and enzyme-linked immunosorbent assay (ELISA) tests.

Experimental dogs were sorted into four experimental groups as follows: (*i*) control group C (*n*  = 5) received 1 mL of sterile 0.9% saline; (*ii*) LB group (*n*  = 5) received 600 µg of *L. braziliensis* promastigote protein in 1 mL of sterile 0.9% saline; (*iii*) Sap group (*n*  = 5) received 1 mg of saponin (Sigma Chemical Co., St. Louis, MO, USA) in 1 mL of sterile 0.9% saline; and (*iv*) LBSap group (*n*  = 5) received 600 µg of *L. braziliensis* promastigote protein and 1 mg of saponin in 1 mL of sterile 0.9% saline. In each case animals received three subcutaneous injections in the right flank at intervals of 4 weeks. Three and a half months (105 days) after the last vaccine dose, the dogs were challenge by the intradermal route in the right ear, with 1×10^7^ late-log-phase *L. infantum* promastigotes (MHOM/BR/1972/BH46) plus SGE obtained by two acini from the salivary glands of *Lutzomyia longipalpis.* Evaluations of humoral and cellular immune response were performed before challenge (Tbc; i.e. 85 days before experimental challenge) and 20, 90, 274, 435, 541 and 885 days after challenge (dac). The dogs were euthanized in 885 dac and tissues were collect to evaluate the cytokine profile and parasite load.

### Blood Sample Collection

Peripheral blood (5 mL) was collected from the jugular vein of each dog and transferred to tubes containing sufficient EDTA to produce a final concentration of 1 mg/mL. The absolute count of lymphocytes in each sample was obtained using a BC-2800 VET auto hematology analyzer (Mindray, China). Blood samples were stored at room temperature for up to 12 h prior to processing.

### Humoral Immune Response

Humoral immune response was evaluated by using soluble lysate of *L. infantum* antigen (MHOM/BR/1972/BH46) (SLcA) according to the conventional ELISA as previously described by Reis *et al*. [29] and Giunchetti *et al*. [23].

### Immunophenotyping and Flow Cytometry

Unlabeled canine monoclonal antibodies (mAbs) anti-CD5 (rat-IgG2a: clone YKIX322.3), anti-CD4 (rat-IgG2a: clone YKIX302.9), anti-CD8 (rat-IgG1: clone YCATE55.9) and anti-MHC-II (rat-IgG2a: clone YKIX334.2), all purchased from Serotec, USA, were used in an indirect immunofluorescence procedure in which pooled normal rat serum (diluted 1∶6000) was employed as the isotypic control and fluorescein isothiocyanate (FITC)-labelled IgG sheep anti-rat polyclonal antibody was used as the secondary antibody. FITC-labelled mouse anti-human-CD21 (mouse-IgG1: clone IOBla) and phycoerythrin-Cy5-conjugated mouse anti-human-CD14 (mouse-IgG2a: clone TUK4) mAbs were used in a direct immunofluorescence assay.

Briefly, microplate assays for immunophenotyping canine whole blood leukocytes in both fresh blood samples and peripheral blood mononuclear cells (PBMCs) obtained after *in vitro* stimulation were carried out according to methods previously described by Giunchetti *et al*. [23] and Reis *et al*. [30]. The results were expressed as the percentage of positive cells within the selected gate for cell surface markers presenting bimodal distribution (CD5, CD4, CD8 and CD21). Semi-quantitative analyses were carried out for the cell surface marker (MHC-II) and the results were expressed as the mean fluorescence channel on a log scale as described by Giunchetti *et al*. [23].

### *In vitro* Assays

For *in vitro* evaluation, PBMCs were isolated from 20 mL of heparinised blood that had been layered onto 10 mL of Ficoll–Hypaque density gradient (Histopaque® 1.077; Sigma Chemical Co.) and centrifuged at 450×*g* for 40 min at room temperature. The separated PBMCs were resuspended in Gibco RPMI1640 medium, homogenized, washed twice with RPMI 1640, centrifuged at 450×*g* for 10 min at room temperature, homogenized and finally resuspended in RPMI 1640 at 10^7^ cells/mL. Briefly, for the mitogenic stimulus assays, 25-µL aliquots of PBMCs (2.5×10^5^ cells/well) were added to triplicate wells together with 25 µL of phytohaemagglutinin (2.5 µg/mL; Sigma-Aldrich Chemie GmbH, Taufkirchen, Germany). Incubations were carried out in a humidified 5% CO_2_ atmosphere at 37°C for 3 (mitogenic-stimulated cultures) or 5 days (antigenic-stimulated cultures). In order to investigate the immunophenotypic features, PBMCs were cultured in 48-well flat-bottomed tissue culture plates (Costar, Cambridge, MA, USA), with each well containing 650 µL of supplemented RPMI medium. Aliquots (50 µL) of PBMCs (5.0×10^5^ cells/well) were added to triplicate wells together with 100 µL of vaccine soluble antigen (VSA) (25 µg/mL) or 100 µL of SLcA (25 µg/mL). Incubation was carried out in a humidified 5% CO_2_ atmosphere at 37°C for 5 days, after which the PBMCs were removed for immunophenotyping according to Giunchetti *et al*. [23].

### RNA Extraction, Reverse Transcription cDNA Synthesis and DNA Manipulations

Total RNA extraction from spleen samples was carried out with SV total RNA Isolation System kit (Promega®, Madison, WI, USA) following the manufacturer’s instructions. Strand cDNAs were synthesised from 1.0 µg of total RNA using the ThermoScript™ RT-PCR System (Invitrogen Brasil, São Paulo, SP, Brazil) with oligodT primers according to the manufacturer’s instructions.

Total genomic DNA was extracted from approximately 20 mg of spleen using Wizard™ Genomic DNA Purification Kit (Promega®, Madison, WI, USA) following manufacturer’s recommendations.

### Primers and Real Time PCR

Primers used to amplify GAPDH gene (AB038240) and cytokines [(IL-10 (U33843), IFN-γ (AF126247), TGF-β1 (L34956), TNF-α (DQ923808)] were designed from sequences deposited in GenBank, with the help of Primer Express 3.0 Software (Applied Biosystems, USA) (Table 1). In order to quantify parasite burdens, primers described by Bretagne et al. [31] to amplify a 90 bp fragment of a single-copy-number gene of DNA polymerase of *L. infantum* (GenBank accession number AF009147) were used. All primers and amplicons are described in Table 1.

**Table 1:** Sequences of primers used for real-time PCR.

Target	Primer sequence (5′–3′)	Product (bp)
GAPDH	Forward: TTCCACGGCACAGTCAAG	115
	Reverse: ACTCAGCACCAGCATCAC	
IFN-γ	Forward: TCAACCCCTTCTCGCCACT	113
	Reverse: GCTGCCTACTTGGTCCCTGA	
TNF-α	Forward: CGTCCATTCTTGCCCAAAC	94
	Reverse: AGCCCTGAGCCCTTAATTC	
IL-10	Forward: AGAACCACGACCCAGACATC	129
	Reverse: CCACCGCCTTGCTCTTATTC	
TGF-β1	Forward: AGGATCTGGGCTGGAAGTG	134
	Reverse: CGGGTTGTGCTGGTTGTA	
*Leishmania*	Forward: TGT CGC TTG CAG ACC AGA TG	90
	Reverse: GCA TCG CAG GTG TGA GCA C	

Cytokines PCR was carried out in a final volume of 25 µL containing 100 mM of forward and reverse primers, SYBR® Green PCR Master Mix (PE Applied Biosystems, Foster City, CA, USA), and cDNA diluted at 1∶5. The samples were incubated at 95°C for 10 min and then submitted to 40 cycles of 95°C for 15 s and 60°C for 1 min, to collect the time fluorescence data. The efficiency of each pair of primers was evaluated by serial dilution of cDNA according to the protocol developed by PE Applied Biosystems. In order to evaluate gene expression, three replicate analyses were performed and the amount of target RNA was normalized with respect to the control (housekeeping) gene GAPDH and expressed according to the 2^−ΔΔCt^ method. The results are expressed as log of mRNA relative expression.

In order to quantify parasite burdens, we used primers that amplified a 90-bp fragment of a single-copy of the gene of DNA polymerase of *L. infantum*. PCR was carried out in a final volume of 25 µL containing 200 nM forward and reverse primers, 1×SYBER GREEN reaction master mix (Applied Biosystems, USA), and 5 µL of template DNA. PCR conditions were as follows: an initial denaturation step at 95°C for 10 min followed by 40 cycles of denaturation at 95°C for 15 s and annealing/extension at 60°C for 1 min. Standard curves were prepared for each run using known quantities of pGEM® T plasmids (Promega, USA) containing inserts of interest [32]. In order to verify the integrity of the samples, the same procedure was carried out for the GAPDH gene (115 bp fragment GenBank accession number AB038240). Reactions were processed and analyzed in an ABI Prism 7500-Sequence Detection System (Applied Biosystems, USA). The results were expressed as the number of amastigotes per mg of spleen.

### Statistical Analysis

Statistical analyses were performed using Prism 5.0 software package (Prism Software, Irvine, CA, USA). Normality of the data was demonstrated using a Kolmogorov–Smirnoff test. One-way analysis of variance and Tukey post-tests were used for determining the differences between groups in terms of humoral immune responses, immunophenotypic profiles, *in vitro* assays at all times evaluated during the *in vitro* longitudinal analyses, cytokines and parasite burden. In all cases, the differences were considered significant when *p* values were <0.05.

## Results

### *Leishmania*-specific Humoral Immune Response in Dogs Vaccinated with LBSap after Challenge with *L. infantum* Plus SGE

**Figure 1:**
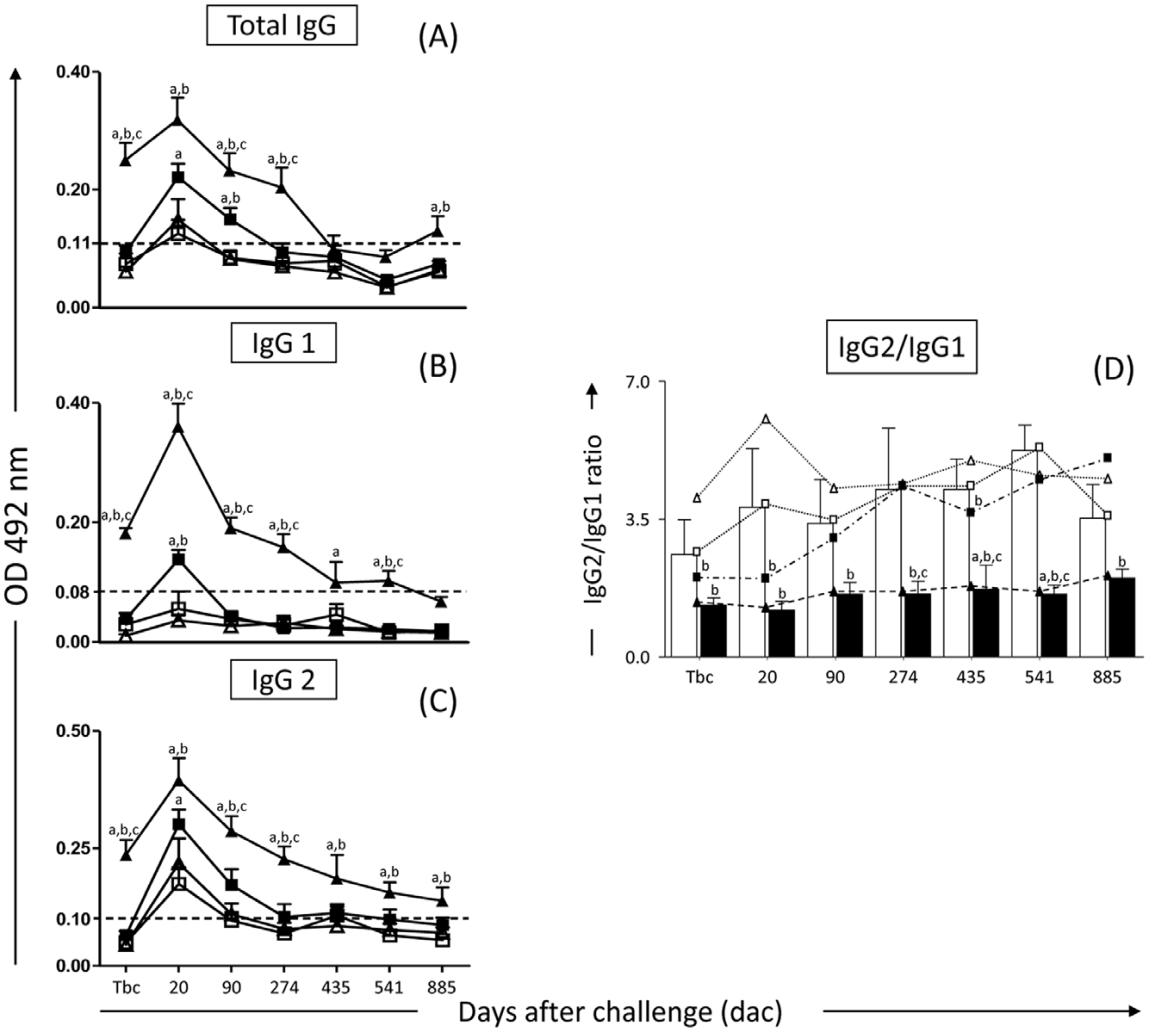
Anti-*Leishmania* reactivity in serum from dogs submitted to different vaccination protocols before and after intradermal challenge with *L. infantum* plus SGE: C (control; white square); Sap (saponin; white triangle); LB (killed *L. braziliensis* vaccine; black square); LBSap (killed *L. braziliensis* vaccine plus saponin; black triangle). IgG2/IgG1 ratio: C (control; white square) and LBSap (killed *L. braziliensis* vaccine plus saponin; black square). Figure 1A represents anti-*L. infantum* total IgG, (B) anti-*L. infantum* IgG1, (C) anti-*L. infantum* IgG2 and (D) IgG2/IgG1 ratio: the *x*-axis displays the times at which the assays were conducted (Tbc: time before challenge with *L*. *infantum*; and 20, 90 274, 435, 541 and 885 days after challenge [dac] with *L*. *infantum*), and the *y*-axis represents the mean ELISA absorbance values determined at 492 nm in serum samples diluted 1∶80 for IgG total and subclasses. The cut-off is represented by the dotted line. Significant differences (*p*<0.05) between the LBSap group and the control C, Sap and LB groups are indicated, respectively, by the letters a, b, and c.

Significant (*p*<0.05) increases in the serum levels of anti-*Leishmania* total IgG (Fig. 1A) at the time before challenge (Tbc; i.e. 85 days before experimental challenge), 20, 90 and 274 days after challenge (dac) were observed in the LBSap group when compared with the C and Sap groups at 20 dac as well as C, Sap and LB groups at Tbc and 90 and 274 dac. LB group showed higher levels (*p*<0.05) of total IgG compared with C at 20 dac and compared with C and Sap dogs at 90 dac (Fig. 1A). Also, the LBSap group elicited higher levels (*p*<0.05) of total IgG at 885 dac compared with those of C and Sap groups (Fig. 1A). Moreover, the LBSap group showed higher levels (*p*<0.05) of IgG1 at Tbc and 20, 90, 274 and 541 dac when compared with the other groups (C, Sap and LB) and compared with C dogs at 435 dac (Fig. 1B). Additionally, at 20 dac, we observed higher levels (*p*<0.05) of anti-*Leishmania* IgG1 in LB dogs compared with C and Sap groups (Fig. 1B). Higher levels (*p*<0.05) of anti-*Leishmania* IgG2 were observed in the LBSap group at Tbc and 90 and 274 dac compared with other groups. Furthermore, at 20, 435, 541 and 885 dac the LBSap dogs showed increases (*p*<0.05) of IgG2 compared with C and Sap groups (Fig. 1C). LB showed higher levels (*p*<0.05) of IgG2 compared with C group only at 20 dac (Fig. 1C).

Further analysis demonstrated that the IgG2/IgG1 ratio was lower (*p*<0.05) in the LBSap group when compared with Sap (Tbc and 20, 90, 274, 435, 541 and 885 dac), C (435 and 541 dac) and LB (274, 435 and 541 dac) groups (Fig. 1D).

### Dogs Vaccinated with LBSap Elicited High Number of Circulating CD5^+^ T Lymphocytes, as well as CD4^+^ and CD8^+^ Subsets, CD21^+^ B Cells and Circulating CD14^+^ Monocytes after Challenged with *L. infantum* Promastigotes Plus SGE

**Figure 2:**
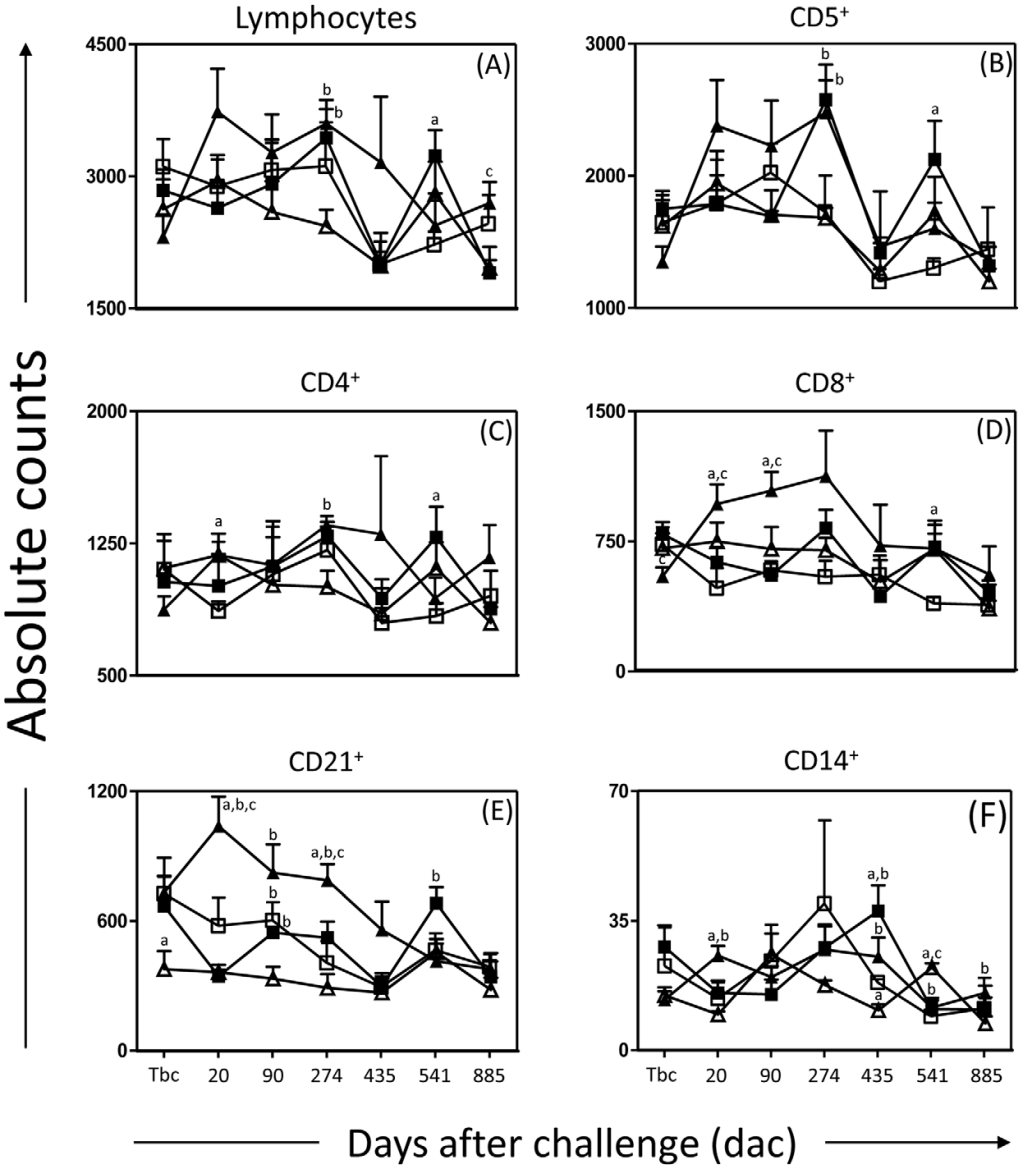
Cellular profile of circulating lymphocytes and monocytes in dogs submitted to different vaccination protocols before and after challenge with *L. infantum* plus SGE. C (control; white square); Sap (saponin; white triagle); LB (killed *L. braziliensis* vaccine; black square), LBSap (killed *L. braziliensis* vaccine plus saponin; black triagnle): the *x*-axis displays the times at which the assays were conducted (Tbc: time before challenge with *L*. *infantum*; and 20, 90 274, 435, 541 and 885 days after challenge [dac] with *L*. *infantum*), and the *y*-axis represents the mean values of the (A) absolute counts of circulating lymphocytes and of (B) CD5^+^, (C) CD4^+^, (D) CD8^+^, (E) CD21^+^ cells and (F) CD14^+^ monocytes. Significant differences (*p*<0.05) between the LBSap group and the control C, Sap and LB groups are indicated, respectively, by the letters a, b and c.

A comparative analysis of the cell profiles of dogs immunized with LBSap and different vaccine formulations showed that the number of circulating lymphocytes increased (*p*<0.05) in LB and LBSap-vaccinated dogs when compared with the Sap group at 274 dac. Also, the LB group showed increase (*p*<0.05) number of circulating lymphocytes at 541 dac compared with C dogs and LBSap group showed significant (*p*<0.05) augmentation of circulating lymphocytes when compared with the LB group at 885 dac (Fig. 2A). In order to evaluate the cellular immunophenotype profile, we enumerated the frequency of T lymphocytes (CD5^+^) and their major subpopulations (CD4^+^ and CD8^+^) (Fig. 2B, C and D). Our results revealed an increase (*p*<0.05) in the number of circulating CD5^+^ T lymphocytes in dogs vaccinated with LB and LBSap compared with the Sap group at 274 dac. Moreover, LB group revealed an increase (*p*<0.05) in CD5^+^ T lymphocytes at 541 dac compared with C dogs.

Analyses of the data showed an increase (*p*<0.05) of circulating CD4^+^ T lymphocytes in LBSap group in comparison with the C and Sap groups at 20 and 274 dac, respectively. Also, the LB group showed increase (*p*<0.05) number of circulating CD4^+^ T lymphocytes at 541 dac compared with C dogs. On the other hand, lower (*p*<0.05) CD8^+^ T-cell counts were observed in dogs vaccinated with LBSap at Tbc compared with the LB group. Additionally, at 20 and 90 dac, we observed an increased (*p*<0.05) number of circulating CD8^+^ T lymphocytes in animals vaccinated with LBSap when compared with C and LB. CD8^+^ T circulating lymphocytes showed increase (*p*<0.05) in the LB group at 541 dac compared with C dogs. In addition, the LBSap group showed a significant increase (*p*<0.05) in CD21^+^ B cells at 20 and 274 dac when compared with C, Sap and LB groups (Fig. 2E). Moreover, LB and LBSap groups revealed an increase (*p*<0.05) in CD21^+^ B cells at 90 dac compared with Sap dogs. LB dogs showed a significant increase (*p*<0.05) in CD21^+^ B lymphocytes compared with Sap group at 541 dac.

We evaluated the levels of antigen-presenting cells (APCs) in dogs that had been vaccinated with LBSap, and observed that the number of circulating CD14^+^ monocytes was higher (*p*<0.05) at 20 dac in this group compared with the C and Sap groups (Fig. 2F). Similarly, LBSap and LB dogs showed higher (*p*<0.05) absolute numbers of CD14^+^ monocytes at 435 dac compared with Sap and C and Sap groups, respectively. In addition, C, LB and LBSap groups showed lower (*p*<0.05) absolute numbers of CD14^+^ monocytes at 541 dac compared with the Sap group. We also observed that the numbers of circulating CD14^+^ monocytes were higher (*p*<0.05) in LBSap-vaccinated dogs at 885 dac as compared with the Sap group (Fig. 2F).

### *In vitro* Cell Proliferation in the Presence of Antigenic Stimuli Increased Intensely in LBSap Dogs after Experimental Challenge

**Figure 3:**
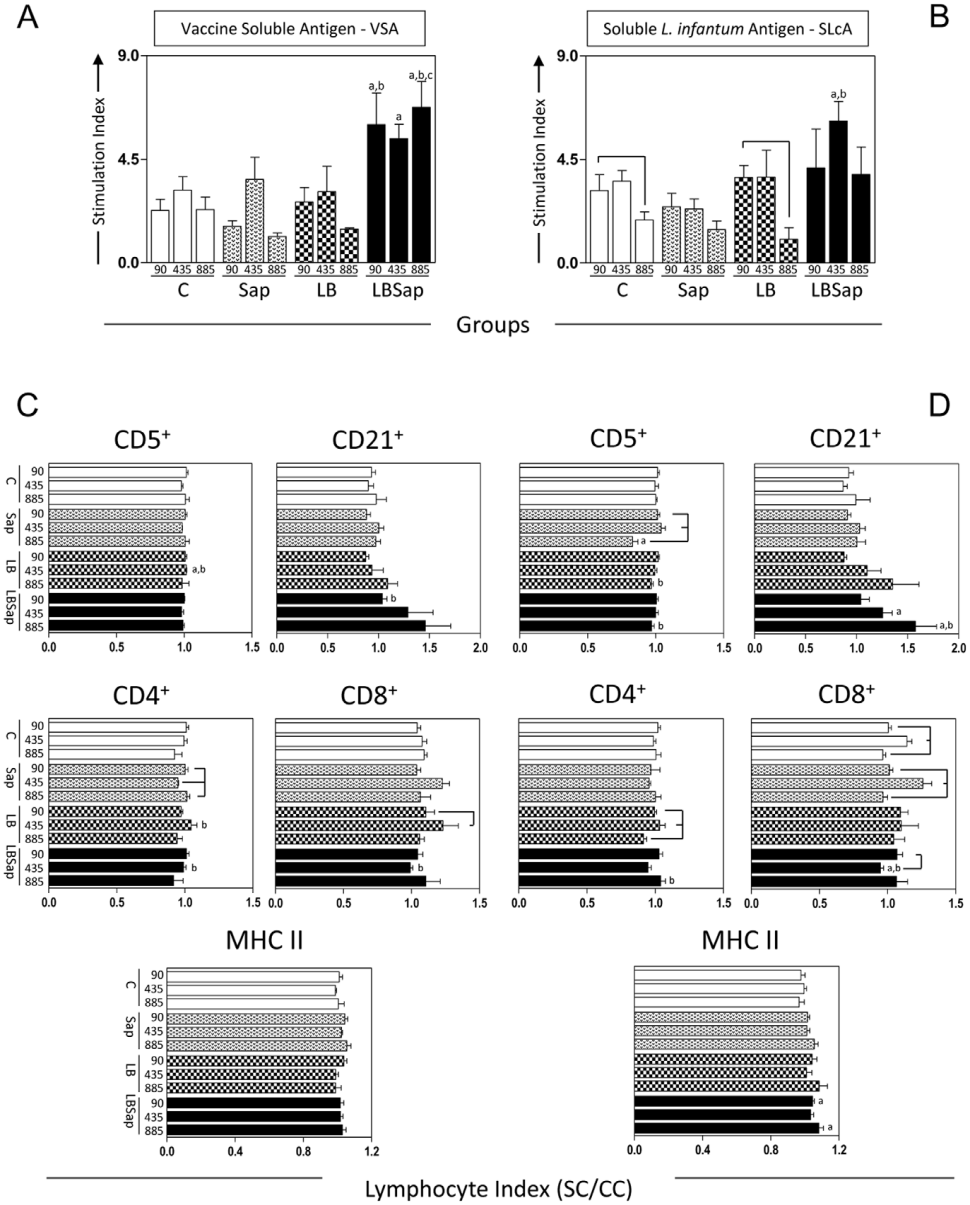
Cell proliferation response of PBMCs after stimulation with (A) vaccine soluble antigen (VSA) and (B) soluble *L. infantum* antigen (SLcA). The middle and lower panels show the immunophenotypic profile of *in vitro* peripheral blood mononuclear cells following stimulation with (C) VSA and (D) SLcA determined at 90, 435 and 885 dac (days after challenge with *L. infantum* plus SGE) for vaccinated groups: C (control; white); Sap (saponin; white with weak hatched); LB (killed *L. braziliensis* vaccine; white with strong hatched); LBSap (killed *L. braziliensis* vaccine plus saponin; black). The results are expressed as index (stimulated cultures over non-stimulated cultures (SC/CC)) of the mean frequencies of CD5^+^, CD21^+^, CD4^+^ and CD8^+^ cells and MHC II expression in lymphocytes (reported as Mean Fluorescence Channel - MFC values). Significant differences (*p*<0.05) between values measured at 90, 435 and 885 dac under the same group are indicated by connecting lines, and between the LBSap and the control C, Sap and LB groups are represented by the letters a, b and c, respectively.

To explore the *in vitro* cell proliferation (PBMCs) we used two different antigenic stimuli: VSA *(L. braziliensis)* in order to evaluate memory lymphoproliferative immune response against antigens of the vaccine components, and SLcA to investigate possible lymphoproliferative homology with the etiological agent of VL (*L. infantum*) (Fig. 3A and B). Comparative analysis of the different treatment groups showed a significantly augmented (*p*<0.05) stimulation index at 90, 435 and 885 dac in the LBSap dogs compared with the C (90, 435 and 885 dac), Sap (435 and 885 dac) and LB (885 dac) groups in VSA stimuli (Fig. 3A). In addition, the LBSap group exhibited a higher (*p*<0.05) lymphoproliferative index at 435 dac compared with the C and Sap dogs after SLcA stimuli (Fig. 3B).

### Higher Frequencies of CD21^+^ B Lymphocytes, CD4^+^ T Lymphocytes and Increased Levels of MHC-II Expression in Antigen-stimulated Cultures Related to Major Phenotypic Changes in Dogs Vaccinated with LBSap and Challenged with *L. infantum*

In order to evaluate whether the phenotypic profile of PBMCs in vaccinated/challenged dogs was influenced by the VSA or SLcA stimulation as well as to characterize these cells, we conducted an analysis of the phenotypic features of PBMCs of vaccinated/challenged dogs (Fig. 3C and D). In the presence of VSA (Fig. 3C), a significant increase (*p*<0.05) in the CD5^+^ T lymphocytes index (stimulated cell/non-stimulated cell) was observed in the LB dogs compared with the C and Sap groups at 435 dac. In addition, the LB and LBSap groups showed a higher (*p*<0.05) CD4^+^ T lymphocytes index when compared with Sap dogs at 435 dac. In contrast, the LBSap group displayed a significant decrease (*p*<0.05) in the CD8^+^ T cells index when compared with the Sap dogs at 435 dac. The CD21^+^ B cells index had a significant increase (*p*<0.05) in LBSap dogs at 90 dac when compared with the Sap group (Fig. 3C).

When we evaluated the SLcA stimulus (Fig. 3D), a significant increase (*p*<0.05) in CD5^+^ T cells index was observed in the LB and LBSap dogs when compared with the Sap group at 885 dac. In addition, the LBSap group showed a higher (*p*<0.05) CD4^+^ T lymphocytes index when compared with the Sap dogs at 885 dac. In contrast, the LBSap group displayed a significant decrease (*p*<0.05) in the CD8^+^ T-cell index when compared with the C and Sap dogs at 435 dac, but an increase (*p*<0.05) at 90 dac when compared with 435 dac in this group. Furthermore, the LBSap group exhibited a higher (*p*<0.05) CD21^+^ B cells index when compared with the C (435 and 885 dac) and Sap (885 dac) groups (Fig. 3D).

We also investigated whether dogs vaccinated/challenged would present PBMCs with different patterns of activation markers. We found that only the LBSap group had an accentuated increase (*p*<0.05) in the MHC-II expression index when compared with the C dogs at 90 and 885dac (Fig. 3D). These findings confirmed that the LBSap vaccine can promote activation of the immune response after challenge with *L. infantum*.

### LBSap Vaccinated Dogs Presenting High Expression of IFN-γ and Low Expression of IL-10 and TGF-β1 in the Spleen Even after 885 Days Post-challenge

**Figure 4:**
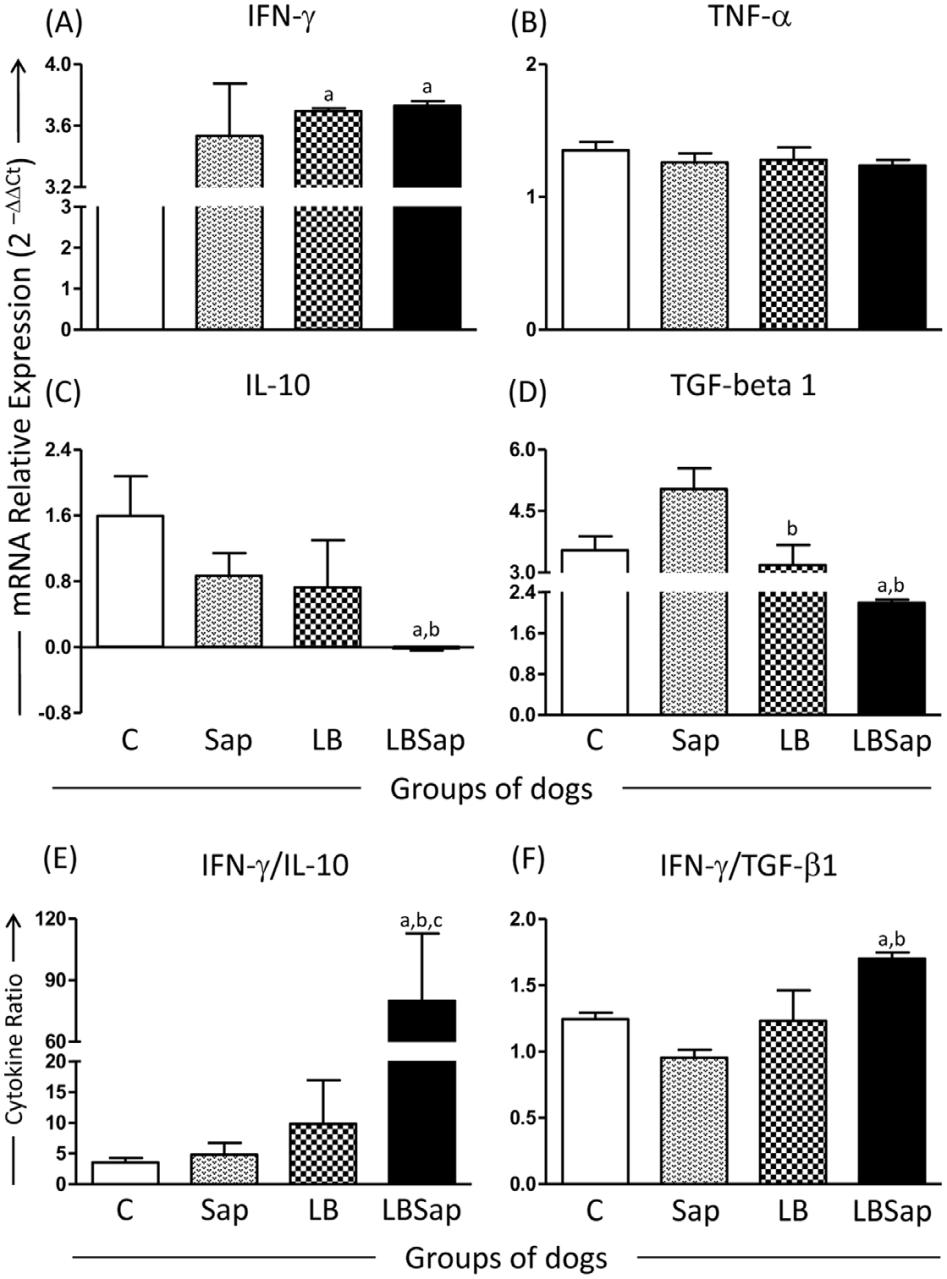
Evaluation of cytokine mRNA levels in spleen samples at 885 dac (days after challenge with *L. infantum* plus SGE) for vaccinated groups: C (control; white); Sap (saponin; white with weak hatched); LB (killed *L. braziliensis* vaccine; white with strong hatched); LBSap (killed *L. braziliensis* vaccine plus saponin; black). The Log number of messenger RNA molecules for (A) IFN-γ, (B) TNF-α (C) IL-10 and (D) TGF- β1 and the cytokines ratio (E) IFN-γ/IL-10 and (F) IFN-γ/TGF-β1 are shown. Results were plotted representing median values for each group. Significant differences (*p*<0.05) between the LBSap and the control C, Sap and LB groups are represented by the letters a, b and c, respectively.

In order to evaluate the profile of immune response in vaccinated/challenged dogs, the genic expression of cytokines was assessed in the spleen of the animals. (Fig. 4). Interestingly, IFN-γ showed higher expression in the LB and LBSap groups when compared with the C group (*p*<0.05). On the other hand, only the LBSap dogs presented lower expression of IL-10 than C and Sap groups (*p*<0.05). Additionally, TGF-β1 was lower expressed (*p*<0.05) in LB and LBSap groups when compared with the C and Sap groups (Fig. 4A).

We also evaluated the IFN-γ/IL-10 or IFN-γ/TGF-β1 ratios in vaccinated/challenged dogs (Fig. 4B). In the spleen, the IFN-γ/IL-10 ratio was higher (*p*<0.05) in LBSap dogs than in the C, Sap and LB groups. Similarly, there was a consistently higher (*p*<0.05) IFN-γ/TGF-β1 ratio in LBSap group than in the C and Sap dogs (Fig. 4B).

### Quantification of Parasite Burden by Real-time PCR

**Figure 5:**
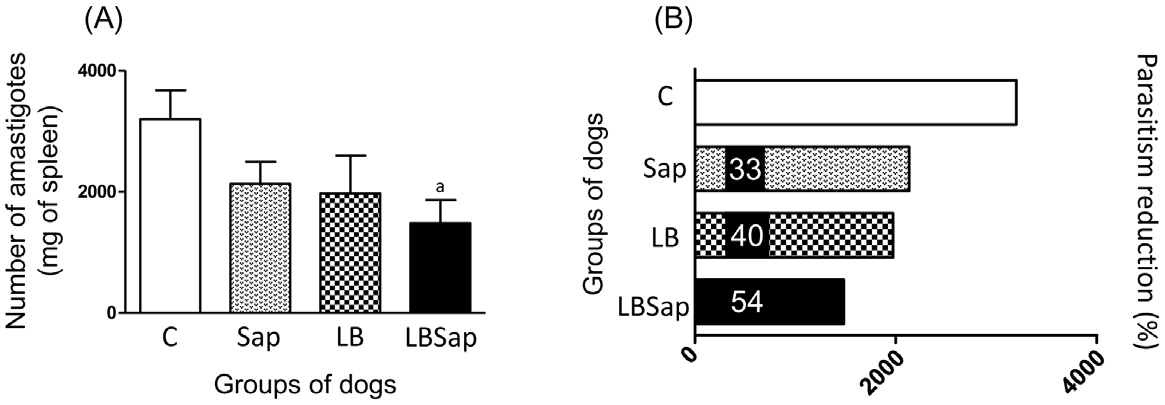
Quantification of parasite burden in spleen samples at 885 dac (days after challenge with *L. infantum* plus SGE) for vaccinated groups: C (control; white); Sap (saponin; white with weak hatched); LB (killed *L. braziliensis* vaccine; white with strong hatched); LBSap (killed *L. braziliensis* vaccine plus saponin; black). (A) The quantification of amastigote forms of *Leishmania/*mg of spleen using real time PCR with specific primers for a single-copy gene of DNA polymerase of *Leishmania infantum*. (B) Parasitism reduction (%) in Sap, LB and LBSap in comparison with the control C group. Results were plotted representing median values for each group. Significant differences (*p*<0.05) between the LBSap and the control C group are represented by the letter a.

The main parasitological features presented by all animals are summarized in Figure 5. As shown in Figure 5A, lower (*p*<0.05) number of amastigotes were observed in LBSap group when compared with C dogs. This data is associated with a parasite burden reduction of 33% in Sap, 40% in LB and 54% in LBSap groups compared with C animals (Fig. 5B). These results indicate the high protective potential of LBSap vaccine even long term after challenge (885 dac).

## Discussion

The increasing expansion and urbanization of VL in Brazil and worldwide [1], [2], show the importance of this emerging and re-emergence disease. The characteristics of this disease and the complexity related to its control are increasingly posing a significant epidemiologic problem. Many efforts are made for identify a profile of biomarkers of resistance or susceptibility in CVL. Increased levels of parameters like parasite load, IL-10 and tumour growth factor-β1 expression, *Leishmania*-specific cell immunodepression or *Leishmania*-specific-IgG, IgM, IgA and IgE serum antibodies are related to clinical disease progression. By contrast, increased levels of parameters such as PBMCs proliferation after leishmanial antigen stimulation, IFN-γ and tumour necrosis factor-α, CD4^+^, CD8^+^ and B cell subsets, or positive leishmanin skin test are related to resistance [33]. The infection in dogs is directly connected to human disease because these animals have certain peculiarities such as high susceptibility to infection, high levels of skin parasites even without clinical signs and its intrinsic connection with the human population [34]. Thus, the development of vaccines against CVL may be an important tool and an effective method to indirectly reduce the incidence of human VL [33], [35], [36], [37]. Numerous anti-CVL vaccines containing diverse antigens and adjuvants have been tested and some have shown promising results [12], [13], [14], [17], [18], [19], [20], [21], [22], [23], [24], [25], [38]. Vaccines derived from whole *Leishmania* antigens are still attractive for use as immunoprophylactics against CVL because they have low-cost production and reduced need for handling and processing of the final product [33], [39] and present promising results for the control of *Leishmania* infection [14], [23], [24], [25], [26], [27].

In the present work we conducted a detailed analysis of the immunogenicity of LBSap vaccine in dogs during 885 days after intradermal inoculation using *L. infantum* and SGE of *L. longipalpis*. The LBSap vaccine has been shown to induce significant immunization effects, including increased Ig isotypes, higher levels of circulating CD8^+^ T lymphocytes, intense cell proliferation, NO production during *in vitro* stimulation and higher levels of *Leishmania*-specific CD8^+^ T lymphocytes [23]. In this work, we observed a strong and sustained induction of humoral immune response in animals immunized with LBSap, with increased levels of total IgG anti-*Leishmania* and their subclasses, IgG1 and IgG2, after experimental challenge. In addition it was observed that the LBSap group has low IgG2/IgG1 ratio values. Molano *et al*. [40], using a recombinant vaccine associated with BCG, obtained similar results. Vaccinated/challenged dogs showed strong serum reactivity with increased production of specific IgG against *L. infantum* antigen. Recently, Fernandes *et al*. [19] demonstrated in a phase II clinical trial, increased production of total IgG and IgG2 in dogs immunized and an increase in IgG1 in non-immunized animals, suggesting that this subclass was associated with a pattern of clinical progression in CVL. On the other hand, other researchers suggest that lower levels of IgG1 are in fact more closely associated with symptomatic dogs [41], [42], [43]. Our results agree in part with those results, describing an association between high levels of IgG1 and the establishment/maintenance of asymptomatic chronic disease as observed in the low IgG2/IgG1 ratio values in the LBSap group. Our findings suggest that the LBSap vaccine promotes a mixed profile of immune responses (Th1/Th2) characteristic of vaccines composed of whole *Leishmania* antigens as reported in previous studies [16], [23], [24], [25], [27].

The intense humoral immune response demonstrated in the LBSap group was synchronous with the increased counts of circulating lymphocytes (274 and 885 dac) and CD21^+^ B cells (Tbc and 20, 90 and 274 dac), probably resulting in the differentiation of plasmocytes and consequent higher levels of Ig secretion. Similar results were observed by Borja-Cabrera *et al*. [18] who demonstrated an increase in CD21^+^ B lymphocytes in a clinical trial after immunization with the Leishmune vaccine. In the natural history of the CVL, Reis *et al*. [44] reported that the decrease in CD21^+^ B cells could be related to increased parasite load in the bone marrow and the severity of clinical signs of the disease. Thus, the increase in CD21^+^ B lymphocytes, characteristic of the group LBSap, may be a biomarker of resistance in the CVL considering this cell as APCs. This hypothesis, however, needs further investigation.

Additionally, protection against *Leishmania* infection depends on the cell-mediated immune response, which implies that an effective vaccine must be able to activate cell-mediated immunity in the immunized animals [33], [45], [46], [47]. In the present study, we observed increased levels of CD4^+^ T cells (20 and 274 dac) as well as CD8^+^ T cells (20 and 90 dac) in the LBSap group. Ramos *et al*. [48] using a DNA vaccine for the LACK protein, did not observe alterations in circulating T-lymphocyte populations (CD4^+^ and CD8^+^) in vaccinated animals after intravenous challenge with *L. infantum*. In a Brazilian endemic area, vaccination using Leishmune® showed increased frequency of CD8^+^ T lymphocytes and absence of CVL clinical signs 18 months after immunization [18]. Indeed, several studies using naturally infected animals showed a correlation between increased numbers of CD4^+^ and CD8^+^ T-cells with resistance against infection by *L. infantum* [33], [44], [49], [50], [51]. Thus, our findings suggest that LBSap-vaccinated dogs present a cell profile that is consistent with the development of a protective immunity against *L. infantum* infection.

In order to evaluate the potential of the vaccine to increase the number of APCs, we measured the levels of circulating CD14^+^ monocytes in all vaccinated dogs after challenge with *L. infantum*. Interestingly, we observed an increase in the numbers of CD14^+^ cells mainly in the LBSap group during the follow-up (20, 435 and 885 dac). Studies on the natural history of the canine disease have shown that symptomatic animals with high parasite loads in the spleen have a dramatic decrease in the circulating CD14^+^ monocyte counts [52]. On the basis of these results, it is possible to speculate that the observed increase of CD14^+^ in LBSap-vaccinated dogs could indicate that a resistance phenotype in the experimental infection with *L. infantum* is related to the levels of these cells, reflecting improved control of tissue parasitism in vaccinated dogs.

To determine whether the LBSap vaccine would activate PBMCs under *in vitro* antigenic stimulation with VSA and SLcA, we measured the stimulation index at 90, 435 and 885 dac in cells derived from immunized dogs. Higher cell reactivity following stimulation by VSA was recorded for the LBSap group at all measured times (90, 435 and 885 dac), whereas stimulation with SLcA showed only higher cell reactivity at 435 dac in the vaccine group. Studies conducted by Lemesre *et al*. [12] showed that an increased cell proliferation rate is directly related to increased production of NO and interferon (IFN)-γ and absence of clinical CVL signs in vaccinated dogs after challenge with *L. infantum*. Similar results were observed by Rafati *et al*. [53] in vaccinated dogs, demonstrating an increase in *in vitro* cell proliferation associated with positive intradermal reaction after experimental challenge. Thus, our findings support the hypothesis that PBMCs from the LBSap group were able to recognize *Leishmania* antigen and proliferate, suggesting that the LBSap vaccine may be a useful tool against CVL.

When *in vitro* cultures of PBMCs derived from the LBSap group were stimulated with VSA or SLcA, increased lymphoproliferation was accompanied by a higher CD5^+^ T lymphocyte (SLcA, 885 dac) and CD4^+^ T cell (VSA, 435 dac; SLcA, 885 dac) index were observed in this group. Following *in vitro* stimulation, a lower CD8^+^ T-lymphocyte index was associated with VSA and SLcA (435 dac) stimulus. Araújo *et al*. [26] showed that dogs immunized with whole parasite vaccine showed an increase in intracytoplasmic IFN-γ on total T lymphocytes (CD5^+^) mainly by the CD4^+^ T-cell subpopulation after stimulation with a specific antigen of *L. infantum*. Taken together, our results suggest that besides the CD8^+^ T-cell population, important in the control of CVL, the CD4^+^ T-cell subset may also simultaneously contribute to improve the cellular immune response in vaccinated dogs to eliminate the parasites from the challenge infection.

In order to evaluate the activation status of lymphocytes in vaccinated/challenged dogs we evaluated the antigen-specific cell profile through the ratio of expression of MHC-II in lymphocytes as previously described [44], [51], [52]. We observed PBMCs from LBSap vaccinated dogs had a higher and sustained MHC-II expression index (90 and 885 dac) in cultures stimulated with SLcA. *In vitro* studies evaluating dogs naturally infected by *L. infantum* showed a decrease in MHC-II expression mainly in symptomatic animals with high parasite burden in the bone marrow and skin [51]. Consistent with these results, it has also been demonstrated that dogs with asymptomatic CVL display enhanced MHC-II expression in circulating lymphocytes (activation status), together with lower overall tissue parasitism [44].

More recently, analyses measuring cytokines (IFN-γ, TNF-α, IL-4, IL-10) in supernatants by ELISA [12], [19], [54] or mRNA expression in by real time PCR [48], [53], [55], [56] have also been used to identify immunological patterns in vaccinated dogs before and after experimental challenge. These studies showed that IFN-γ is a high-quality biomarker of immunogenicity and protection against *Leishmania*. On the other hand, increased IL-10 expression has been associated with increase in parasitic loads and progression of the disease [57], [58]. In this study, we observed increased IFN-γ expression in LB and LBSap groups in the spleen. Interestingly, we observed decreased expression of regulatory cytokines, such as IL-10 in LBSap dogs and TGF-β1, in LB and LBSap groups. These findings reinforce the protection profile of LBSap vaccine. When we evaluated the IFN-γ/IL-10 or IFN-γ/TGF-β1 ratios, we observed that only LBSap group showed higher ratios than the other groups. Overall, our results suggest that dogs vaccinated with LBSap present valuable biomarkers of immunogenicity and protection against canine leishmaniasis even after 885 days post-challenge.

In the context of experimental challenge in the canine model, there are no reports of standardization of the inoculum. The *in vitro* culture passages and the varying concentrations of the parasite used, particularly in vaccine trials [33], [59], have not been shown to be effective in infection and most dogs remain asymptomatic [59], [60]. Furthermore, in this work, we used SGE associated with the experimental challenge trying to mimic the event when the sand fly makes the blood meal and inoculates saliva and *Leishmania* promastigotes. Ours results showed that all dogs remained asymptomatic throughout the follow-up (885 dac) in this study. Thus, the use of SGE was not sufficient to replicate the bite environment.

In the natural history of CVL, the progression of clinical disease is associated with establishment of large numbers of parasites in different tissues [51]. However, the amount of *Leishmania* DNA found in the bone marrow might be significantly higher than that measured in blood samples, in both experimental and natural infections [60]. In this study we selected spleen as the target tissue for detection of the parasite because it is one of the major site for the parasites [44]. Nowadays, the real-time PCR technique has also been used by several authors in order to diagnose and screen the evolution of VL in quantifying the parasite burden presenting with high sensitivity, accuracy and reproducibility [31], [57], [61]. In our work, we applied real-time PCR technique to quantify the number of amastigotes per mg of spleen and we observed that only LBSap group showed lower parasitism. Furthermore, the LBSap dogs showed higher proportion of parasite reduction (54%) compared with LB (40%) and Sap (33%) groups. Thus, the protection obtained in the present study confirms the capacity of LBSap vaccine to limit parasite replication and prevent severe disease even long term after challenge (885 days).

In conclusion, this study demonstrated that immunization with LBSap vaccine is highly immunogenic and demonstrates a persistent humoral and cellular immune response indicating a resistance phenotype against canine *L. infantum* infection. Immunizing dogs with LBSap vaccine resulted in the induction of high levels of total IgG (IgG1 and IgG2); higher levels of lymphocytes, particularly in circulating CD21^+^ B cells and CD4^+^ and CD8^+^ T cells; and important cell proliferation with VSA-SLcA-specific CD4^+^ T lymphocytes.

Furthermore, LBSap vaccinated dogs presented high IFN-γ and low IL-10 and TGF-β1 expression in spleen with significant reduction of parasite load in this tissue. Overall, our results validate the potential of LBSap vaccine to protect against *L. infantum* experimental infection and strongly support further evaluation of efficiency of LBSap against CVL in natural infection conditions.
